# A macrophage attack culminating in microthromboses characterizes COVID 19 pneumonia

**DOI:** 10.1002/iid3.482

**Published:** 2021-07-07

**Authors:** Brian S. Bull, Karen L. Hay

**Affiliations:** ^1^ Department of Pathology and Human Anatomy School of Medicine Loma Linda University Loma Linda California USA

**Keywords:** blood tissues, human animals, infections, viral retroviral

## Abstract

**Introduction:**

A neutrophilic infiltrate characterizes bacterial pneumonia. Macrophage infiltration is similarly characteristic of the viral pneumonia caused by SARS‐CoV‐2. These infiltrating macrophages, while phagocytic and capable of engulfing virus laden alveolar cells, are also rich in tissue factor—a thromboplastin. This prothrombotic aspect likely explains how a respiratory virus whose malign effects should be confined to the oropharynx, bronchi and lungs, can cause a panoply of extra‐pulmonary organ disorders. Elevated ferritin levels in ICU Covid 19 patients, and elevated acute phase proteins suggest immune overreaction. Elevated d‐dimers implicate clotting as well. This evidence links hyperactive innate immunity (macrophage lung infiltrates) with the elevated levels of oligomeric fibrin present in the bloodstream of these patients.

**Methods:**

An in‐house assay measuring oligomeric (soluble) fibrin (also referred to as soluble fibrin monomer complexes or SFMC) in whole blood, previously developed for monitoring incipient disseminated intravascular coagulation (DIC) during liver transplantation, was made available to COVID ICU attendings. Since SFMC constitutes the input to intravascular fibrin clots and d‐dimer reflects fibrin clot dissolution, it was thought that the two tests, run in tandem along with assays of immune activation, might clarify the frequency and possibly the cause of DIC in patients with severe COVID‐19 pneumonia.

**Results:**

Classical DIC with intravascular clotting and thrombocytopenia was documented only rarely. However, early in the pandemic shortly after the assay was made available, it identified three patients undergoing acute defibrination. In each patient virtually all of the body's fibrinogen was transformed into SFMC over 3–4 days and deposited somewhere in the vasculature without any gross clots being detected.

**Conclusions:**

Three COVID‐19 patients with evidence of a hyperactive immune response (elevated ferritin and acute phase proteins) defibrinated while blood levels of SFMC were being monitored. SFMC levels that were five times higher than normal appeared in the circulation during the defibrination process. SFMC at these levels may precipitate as showers of microclots, damaging heart, kidney, brain, and so forth.

## Graphical abstract

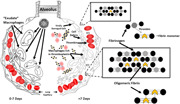

## 1 INTRODUCTION

How is it possible for a *respiratory* virus to damage heart, liver, kidneys, and brain, precipitate diarrhea and produce skin lesions that look like frostbite? In newspaper accounts, COVID‐19 has been associated with each of these findings.^1^ In the formal scientific literature it is reported that this virus can damage the hematologic, cardiovascular, renal, gastrointestinal, hepatobiliary, endocrinological, dermatological, neurological and ophthalmic organ systems.^2^

## 2 COVID PATHOPHYSIOLOGY: A PROPOSAL

We propose that, in severely ill patients, SARS‐CoV‐2 produces these diverse, malign outcomes because the macrophages that respond to infection by this respiratory virus are pro‐coagulant and partially expropriate the clotting cascade.

Cell mapping has confirmed that macrophage infiltration is a defining characteristic of COVID‐19 pneumonia. The unusual nature of this particular pneumonic infiltrate has been tersely epitomized by the following: “Neutrophil and macrophage infiltration are hallmarks of bacterial pneumonia and COVID‐19, respectively.”^3^

Several days into severe COVID‐19 pneumonia, “exudate” macrophages that have assembled in response to a SARS‐CoV‐2 infection of alveolar lung epithelium attack and destroy the virally infected cells. Such infiltrating macrophages are known to be rich in tissue factor (TF).^4^ TF is a trans‐membrane glycoprotein that functions as a cofactor with Factor VII to activate extrinsic clotting. In mouse models of lethal influenza virus pneumonia, this “attack of the macrophages” reaches maximum on day 8 of infection—6 days after the influenza virus titer has peaked.^5^ The “attack of the macrophages” is therefore, not a direct response to viral overload.

However, in COVID‐19 pneumonia, once TF enriched macrophages attack alveolar walls, hemorrhage occurs.^6^ Such hemorrhage occurs into a now thromboplastin‐rich environment. Intra‐alveolar clotting will rapidly follow. In COVID‐19 patients, microscopic clots occur throughout the body.^7^, ^8^, ^9^ It is not unreasonable to assume that this procoagulant production in the alveoli and the occlusion of capillary networks throughout the body by tiny clots, are related as to cause and effect. Most often, the presence of these extra‐pulmonary clots will not be detectable during life. That they exist, however, has been confirmed in living, ventilator‐dependent, COVID‐19 ICU patients.^10^ Eleven of 13 such patients (85%) demonstrated evidence of sublingual microvascular thrombi, with 31% showing completely stagnated capillaries. In three of these patients, an abrupt thromboembolic obstruction was captured on video as it occurred.

**Figure 1:**
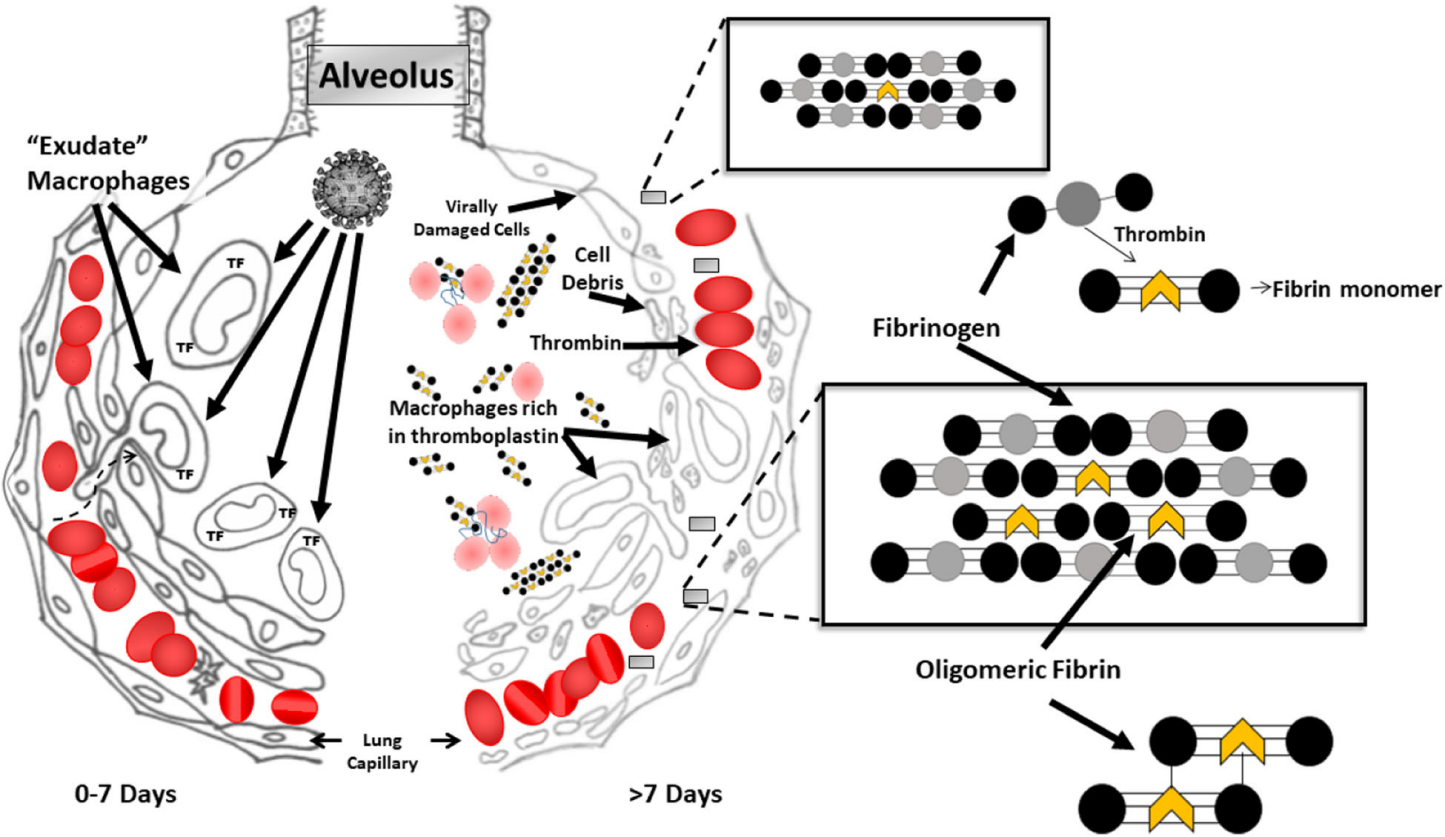
A drawing of the intra‐alveolar sequence of events during COVID‐19 pneumonia. The left half depicts events during the early phase of disease when the virus is multiplying in alveolar lining cells with a response to the infection from “exudate macrophages.” The right half depicts events after the gathered macrophages have attacked the alveolar lining cells, intra‐alveolar hemorrhage has occurred with clotting and thrombin is being infused back into the pulmonary circulation. The blow‐up panels showing molecular structure depict the fibrin‐fibrinogen complexes in the pulmonary capillaries that are now free to circulate systemically

From this point on, the pathophysiology of COVID‐19 pneumonia is no longer primarily that of a viral disease; it is now largely that of a clotting disorder and we can confirm that quantities of molecular aggregates between short‐chain fibrin and native fibrinogen molecules known as soluble fibrin monomer complexes ^11^ (SFMC) appear in the circulation of such patients. We believe it to be the case that, in COVID‐19 patients, SFMC forms primarily in the lungs and then travels throughout the body as illustrated in Figure 1.

If the process continues, increasing levels of SFMC entering the general circulation from the lungs have the potential to produce the microclots that occlude vessels throughout the body. As short‐chain fibrin molecules lengthen by polymerization, they become too long to remain in solution. It is microclots of this size—on the boundary between soluble and insoluble fibrin—that first appear. These microclots are far too small to be detected by routinely available, noninvasive, diagnostic techniques. Thus, their formation, transitory existence, and dissolution will go unobserved. A d‐dimer rise in response is likely to be the only evidence of their transient existence, as SFMC blood levels are only rarely monitored

## 3 A CLINICAL ANALOGUE OF DEFIBRINATION

SFMC will form in response to procoagulant cell debris that gains access to the general circulation. A clinical situation in which this takes place is the disseminated intravascular coagulation—disseminated intravascular coagulation (DIC)—that can develop in patients during liver transplantation surgery. SFMC levels often increase just after reperfusion of the transplanted liver when residual dead and dying cells from the donor liver are washed into the recipient's circulation. (This is occasionally and catastrophically accompanied by the sudden appearance of large clots in the heart and great vessels.) If the newly transplanted liver is healthy and begins functioning immediately, the SFMC levels will generally drop back towards the normal range over the next 60 min or so.

Early in the course of the pandemic, an assay (developed for monitoring for SFMC production during liver transplantation) was made available to intensivists in the COVID‐19 ICU. We report on three patients who developed extremely high levels of SFMC shortly after the assay became available. In each patient, virtually all of the body's fibrinogen was transformed into SFMC over a 3‐ to 4‐day period and deposited somewhere in the vasculature. We report measurement of these oligomeric fibrin molecules and document that the process of SFMC formation may result in whole body defibrination without macroclots being identified clinically.

## 4 MATERIALS AND METHODS

A common daily protocol for laboratory data on COVID‐19 patients with or without ventilator assistance in an ICU ward specifies the measurement of Fibrinogen, d‐dimer, sedimentation rate, ferritin, and C reactive protein. In our Medical Center the protocol was amended to include the option of SFMC measurement should the attending physician consider it warranted.

### 4.1 Precipitation technique and measurement of SFMC—reported as soluble fibrin units (SFU)

Quantification of SFMC was performed as previously described.^12^ Briefly, citrated whole blood was drawn by trauma‐free venipuncture or through a central line after appropriate clearing. Samples were at all times maintained at 37°C. Testing was performed by adding 150 µl well‐mixed whole blood to 450 µl pre‐warmed protamine sulfate reagent (0.4 mg/ml), subjecting the mixture to controlled turbulence (a complex rocking/rolling motion) and determining the time in seconds to first appearance of precipitated SFMC as gel or as clumps. Raw times were then converted to arbitrary soluble fibrin units (SFU) and the amount present expressed in sec^−1^ by dividing the time‐to‐appearance into 700. In our normal population, the reference range is 0–9 SFU for males; 0–11 SFU for females. SFU levels up to ~40 SFU reflect an increasing load of still‐soluble oligomeric fibrin. However, as levels approach 50 SFU in anticoagulated blood on the laboratory bench, microclots begin to form. SFMC levels in normal and patient blood samples are stable for up to 4 h provided the temperature of the sample, throughout the entire period between phlebotomy and testing, is maintained at 37°C. SFMC is partially cold‐precipitable and elevated levels can drop markedly if the blood sample is allowed to cool between specimen acquisition and analysis. Rewarming the specimen to 37°C does not resolubilize the precipitated SFMC.

Protamine sulphate is a strongly positively‐charged protein. When added to whole blood containing SFMC, it weakens the bonds between the fibrin monomers/oligomers and the fibrinogen molecules with which they are complexed.^13^ The mechanical action to which the blood sample is subjected in the SFMC analyzer (and the hydraulic stress that results) potentiates further polymerization of the newly freed up fibrin oligomers until the polymers grow too large to remain in solution. The higher the concentration of SFMC in the sample, the more rapidly the precipitate appears. The endpoint for this assay is defined as the first appearance of fibrin gel or clumps ≥0.5 mm in diameter.

### 4.2 Measurement of SFMC levels and the usefulness of such data

This clinical laboratory assay^12^ that measures oligomeric fibrin levels is a laboratory developed test (LDT)^14^ as defined by the food and drug administrations (FDA's) draft guidance document. The liver transplantation surgeons in our medical center needed an assay that would warn them of impending DIC before they were confronted with gross clots in the heart and great vessels or, alternatively, massive bleeding. None of the available methods took into account the cold sensitivity of the parameter under measurement,^15^ nor were any of the then extant laboratory assays rapid enough (≤ 15 min) to provide useful estimates of SFMC levels to the surgeons while they were transplanting a liver.^16^ To address surgical needs and overcome deficiencies with available tests, we developed the Rapid SF assay. During surgery, it is performed at 60‐min intervals in the transplant surgery suite on whole blood samples maintained at body temperature. Testing is performed immediately following sample collection without any additional specimen processing.

## 5 RESULTS

### 5.1 SFMC levels in three COVID‐19 pneumonia patients

Table 1 summarizes the anticoagulation regimen and pertinent laboratory test results on three COVID‐19 patients in the intensive care unit. Permission for use of this anonymized data was obtained from the Loma Linda University Institutional Review Board. Two of these three patients are now deceased, both dying in multiorgan failure. As of the time of writing, one remains alive—more than 11 months postinfection—but he has suffered brain damage (multiple small infarcts of the basal ganglia), is confined to a walker, and breathes via a tracheostomy.

**Table 1:** Laboratory test results and anticoagulation regimen on three COVID‐19 ICU patients

		Soluble fibrin (SFU)	Fibrinogen (mg/dL)	d‐Dimer (uG/mL)	Ferritin (ng/mL)	Anticoagulation		
Patient #	COVID Day #	M 0‐9F 0‐11	M/F 200‐393	M/F 0‐0.4	M 12‐350F 12‐300	Pre/concurrent with admission	Prophylactic LMW heparin	Therapeutic unfractionated heparin
**#1**	**10**	**<2.3**	686	1.3	3093		40 mg	
	**11**	**Not tested**	470	2.8	2934		40 mg	
	**12**	**Not tested**	312	>21	3314		40 mg	
	**13**	**Not tested**	114	>21	3016		40 mg x 2	
	**14**	**42.4**	76	>21	2807		40 mg	
	**15**	**28.6**	Not tested	>21	2765		40 mg	
	**16**	**15.6**	Not tested	>21	2484		40 mg	
**#2**	**7**	**8.2**	686	2.3	5775		40 mg	
	**8**	**18**	707	3.8	5282		40 mg	
	**9**	**45.8**	539	>21	4793		40 mg	
	**10**	Not tested	Not tested	Not tested	4122			
	**11**	**24.1**	178	19.2	41990		40 mg	
	**12**	Not tested	Not tested	Not tested	16082			
	**13**	**28**	89	>21	8159			5000 U inj x2
**#3**	**4**	**<2.3**	514	6.3	3759	Coumadin(INR 2.8)		
	**5**	**51.1**	526	>21	3336	Coumadin(INR 3.2)		
	**6**	**52.6**	236	>21	3611	Coumadin(INR 7.9)		
	**7**	**53.0**	186	>21	2376	Coumadin(INR 14.8)		
	**8**	**47.3**	156	>21	1971			7000 U injIV drip started
	**9**	**21.1**	164	19.2	1792			1000 U/h
	**10**	**<2.3**	285	6.6	1599			600 U/h

The pattern of the laboratory values as each case progressed is interesting and possibly instructive. It is unquestionable that all three patients underwent defibrination over ~4 days. That defibrination occurred is evidenced by the decrease in fibrinogen levels, with fibrinolysis confirmed by the d‐dimer results. Fibrinogen levels in patient #1 decreased from 686 to 76 mg/dl between days 10 and 14 of COVID‐19 pneumonia. In patient #2, fibrinogen dropped from 707 to 89 mg/dl between days 8 and 13. Patient #3 defibrinated over a similar time period while INRs ranged from 2.8 to 13.8. d‐dimers, on most days while defibrination was progressing in all three patients, exceeded the upper limit of the laboratory's reportable range (>21.0 µg/ml). This level is ˜50x the upper end of the reference range for this assay (0.4 µg/ml).

All three patients showed extremely high ferritin levels (reference range 12–350 ng/ml). This finding is common in COVID‐19 patients with moderate to severe pneumonia. Ferritin is an acute‐phase protein, and it likely also reflects the activity of activated macrophages.^17^

SFMC levels in patient #1 were below detection limits on the day of admission (day 10 of illness). SFMC levels this low are common in patients receiving “prophylactic” heparin (i.e., 40 mg Lovenox q.d.). This patient was prophylactically anticoagulated with Lovenox on the day of admission. Given the undetectable SFMC levels that were the result of this minimal anticoagulation, no further assays were ordered until day 13. By that time, defibrination had occurred and fibrinogen had dropped from 686 to 76 mg/dl.

Patient #3 was not given heparin on admission as this patient was fully anticoagulated with long‐term Coumadin (INR 2.8) for a coexisting cardiovascular condition. Considering the attendant procoagulant threat of vitamin K administration and/or of Factors II, VII, IX and X transfusions (and that the patient was already fully anticoagulated), Coumadin was not reversed and no heparin was given. As can be seen from subsequent laboratory data, therapeutic, and supra‐therapeutic INRs did not inhibit the generation of SFMC in this patient. On Day 8, in view of the persistently high levels of d‐dimer and SFMC, Vitamin K was administered and the patient was placed on a heparin drip. Following these actions, the defibrination process slowed significantly.

## 6 DISCUSSION

The fact that SFMC is mostly soluble does not mean it is innocuous. If fragile microclots are forming and dissolving throughout the circulation, then fragile microclots may *temporarily* occlude random portions of the microvasculature throughout the body. The behavior of these delicate early microclots is likely influenced by two different, but well studied, mechanisms. (1) SFMCs, protofibrils and early fibrin microclots are much more dynamic than was first realized. Before Factor XIII‐induced crosslinking, monomers/oligomers can rapidly dissociate from or insert themselves into other developing fibrin structures. If blood flow is rapid enough, dissociated fibrin monomers/oligomers will be carried away, thereby potentially reducing the developing blockage.^18^ (2) Plasmin‐induced fibrinolysis can rapidly break down both microclots as well as clots formed in the presence of a paucity of thrombin.^19^ In either case there will be scant or absent x‐ray and autopsy evidence that clots were ever present.

Such transiently‐present microclots would account for many, if not most, of the bizarre manifestations of COVID‐19—both during the acute phase of the disease itself and, in some unfortunate patients (the ‘Long‐COVID' syndrome sufferers), for weeks or months afterwards. These manifestations would be the delayed effects of widespread—even if largely temporary—microvascular occlusions. Most of these micro‐occlusions will likely dissipate after only a transitory existence; however, those that do not immediately dissipate will cause coagulation necrosis and death of the tissue downstream.

It is clearly quite possible for a *respiratory* virus to damage heart, liver, kidneys, brain, bring on diarrhea and cause skin lesions that look like frostbite if that virus, by coopting the clotting system, is capable of generating large quantities of SFMC in the bloodstream. If those fibrin molecules are of short chain‐length only, and if they are complexed to native fibrinogen so that they remain in solution, they have access to all parts of the body. Whether the virus itself travels along with the oligomeric fibrin/fibrinogen complexes remains to be determined. We think that it is unlikely that a virus, optimized for replication in respiratory epithelium, is capable of infecting and proliferating in the plethora of cell types that are affected in COVID‐19 patients. Viral particles, though, may well be detected in the debris from virally damaged alveolar lining cells that have entered the circulation. Elevated SFMC levels, however, are certainly capable of damaging all cell types in the body via sporadic occlusion of portions of the microcirculation.

This atypical clotting is, very likely, the result of minute quantities of thrombin entering the bloodstream again and again—for repeatedly introducing minute quantities of thrombin or thromboplastin into vigorously stirred, anticoagulated blood does produce fibrin oligomers of short enough chain length that they remain soluble (data not shown). From this evidence it seems likely that, as each alveolus succumbs under COVID‐19 attack, a minute amount of thrombin initiated by TF‐rich macrophages and other cellular debris will gain access to the pulmonary circulation.

In severe COVID‐19 pneumonia, dead and dying alveolar cells along with TF‐rich macrophages fill damaged alveoli and infiltrate pulmonary tissue. This infiltrate, being rich in TF can activate the extrinsic clotting pathway on contact. (Figure 1) Should hemorrhage into the damaged alveolus occur (at autopsy, alveolar hemorrhage is widespread)^6^, ^7^ all of the elements for activation of extrinsic clotting and the production of SFMC are in place. However, instead of a massive infusion of dead and dying cells as may occur during liver transplantation, this thrombin generation and/or infusion of necrotic cells will occur one dying alveolus at a time.

The anomalous variant of clotting that occurs in SARS‐CoV‐2 infected lungs can now extend its damaging effects to the entire body through production of SFMC. When short chains of oligomeric fibrin encounter one another they will polymerize, lengthen and form two‐stranded protofibrils. These encounters become more likely due to circulatory conditions such as cooling in the extremities, vascular narrowing, roughened endothelium or other factors that result in turbulence. Once protofibrils reach ~20–25 monomers (0.5–0.6 µm) in length or longer they will no longer be soluble.^18^, ^20^ As the protofibrils lengthen they become long enough to self‐interact and aggregate laterally. A sol to gel transition occurs and clots form.^21^, ^22^

Finally, full blown defibrination occurred in in one of the patients despite supra‐therapeutic INR levels. This raises the possibility that, in COVID 19 coagulopathy, anti‐Vitamin K therapy may not be adequate. It likely also implies that heparin's effectiveness is not solely due to its anticoagulant properties. Heparin potentiates the release of TF Pathway Inhibitor (TFPI).^23^

High levels of SFMC exist in the blood of severely ill COVID‐pneumonia patients. If these levels were routinely monitored they could alert clinicians when pulmonary infiltration by thromboplastin‐rich macrophages is about to transform a viral infection of the lower respiratory tract into a consumptive coagulopathy/viral pneumonitis.

## CONFLICT OF INTERESTS

Both authors are holders of a patent (now expired) for an original version of the nonautomated instrument that, among other assays, measured SFMC. Both authors have filed for patent protection on an instrument, under development, that is capable of performing similar assays in fully automated mode.

## AUTHOR CONTRIBUTIONS

Both authors contributed to project conception, project development, acquiring background information, laboratory experiments, writing and editing this paper.

## Supporting information

Supporting information.
